# DeepAD: A deep learning application for predicting amyloid standardized uptake value ratio through PET for Alzheimer's prognosis

**DOI:** 10.3389/frai.2023.1091506

**Published:** 2023-02-06

**Authors:** Sucheer Maddury, Krish Desai

**Affiliations:** Leland High School, San Jose, CA, United States

**Keywords:** Alzheimer's disease, PET, amyloid, convolutional neural network, gradient boosted decision tree

## Abstract

**Introduction:**

Amyloid deposition is a vital biomarker in the process of Alzheimer's diagnosis. ^18^F-florbetapir PET scans can provide valuable imaging data to determine cortical amyloid quantities. However, the process is labor and doctor intensive, requiring extremely specialized education and resources that may not be accessible to everyone, making the amyloid calculation process inefficient. Deep learning is a rising tool in Alzheimer's research which could be used to determine amyloid deposition.

**Materials and methods:**

Using data from the Alzheimer's Disease Neuroimaging Initiative, we identified 2,980 patients with PET imaging, clinical, and genetic data. We tested various ResNet, EfficientNet, and RegNet convolutional neural networks and later combined the best performing model with Gradient Boosting Decision Tree algorithms to predict standardized uptake value ratio (SUVR) of amyloid in each patient session. We tried several configurations to find the best model tuning for regression-to-SUVR.

**Results:**

We found that the RegNet X064 architecture combined with a grid search-tuned Gradient Boosting Decision Tree with 3 axial input slices and clinical and genetic data achieved the lowest loss. Using the mean-absolute-error metric, the loss converged to an MAE of 0.0441, equating to 96.4% accuracy across the 596-patient test set.

**Discussion:**

We showed that this method is more consistent and accessible in comparison to human readers from previous studies, with lower margins of error and substantially faster calculation times. We implemented our deep learning model on to a web application named DeepAD which allows our diagnostic tool to be accessible. DeepAD could be used in hospitals and clinics with resource limitations for amyloid deposition and shows promise for more imaging tasks as well.

## 1. Introduction

Alzheimer's disease is a worldwide health concern which has many neurological effects. This common neurological disorder results in brain atrophy, causing patients to experience cognitive decline, behavioral change, and memory loss (Lane et al., 2018). Diagnosis (particularly early diagnosis) for Alzheimer's is imperative in order to implement proper treatment plans and delay the progression of the disease (Rasmussen and Langerman, 2019). Efficient and accurate diagnosis is also important in order to save time and reduce error. There is also an overlap in what doctors consider abnormal change and normal age-related change (Mayo Clinic Staff, 2022); this creates assessment variability which is an inconsistent practice.

Imaging, clinical data, and physiologic biomarkers are major factors in AD prognosis. Positron Emission Tomography (PET) is an imaging technique which provides 3D images that can be used to quantify biochemical processes in the brain (Passamonti et al., 2017). The radiopharmaceutical Florbetapir (18F-AV-45) traces amyloid deposition, an important biomarker which correlates to the progression of Alzheimer's disease (King-Robson et al., 2021).

The Standard Uptake Value Ratio (SUVR) is commonly used as a quantitative measurement of the radiotracer uptake in the brain (Vemuri et al., 2016). Pre-existing SUVR values calculated from ^18^F-florbetapir PET imaging scans were used in our model. To streamline the process of calculating SUVR, we used novel deep learning architecture, a powerful tool that improves efficiency and accuracy in Alzheimer's prognosis (Saleem et al., 2022). We combined deep learning architecture, which was optimized for linear regression, and gradient boosted decision trees to create a SUVR prediction model for this analysis.

## 2. Background literature

Extracellular amyloid plaques are important in AD characterization (Bloom, 2014). Amyloid-β (Aβ) peptides, derived from the amyloid beta precursor protein, are made from amyloid plaques. The accumulation of amyloid plaques disrupts the synapses that facilitate cognition and memory, which show that amyloid beta accumulation is a hallmark of AD. Also, the accumulation of Aβ amyloid fibrils lead to tau synaptic dysfunction which is more indicative of cognitive and memory loss in AD subjects compared to Aβ (Bloom, 2014).

Several studies have examined the relationship between amyloid and AD pathology. Biomarkers use parameters to measure the presence of a disease in a patient. Camus et al. (2012) determined that Florbetapir (18F-AV-45) is a core radiotracer biomarker for AD which binds to amyloid plaques. This study found that the mean quantity values of SUVR were higher in AD subjects than HC (Healthy Controls) subjects in cortical regions when using ^18^F-florbetapir. Because ^18^F-florbetapir tracers selectively bind to amyloid in human brain tissue (Choi et al., 2012), the higher cortical uptake of ^18^F-florbetapir in MCI and AD subjects compared to HC subjects show that there is a strong correlation between amyloid and AD pathology.

SUVR is a common way to quantify the severity of a disease. Vemuri et al. (2016) wrote that SUVR is a semi-quantitative measurement which is calculated by the uptake of a radiotracer with respect to the reference region. SUVR can be measured with the uptake values of the ^18^F-florbetapir radiotracer. Kinahan and Fletcher (2010) quantified SUVR as the radioactivity concentration from the radiotracer in the region of interest (ROI) averaged over the cortical and subcortical regions divided by the reference tissue activity over the same period used to calculate the standard uptake value.

Although studies indicate that an accumulation of amyloid-β corresponds to the characteristics of AD pathology, Ingeno (2019) showed that the removal of amyloid from the brain resulted in the same or worsened cognitive state when performing clinical trials. However, data on amyloid-β can be utilized for AD prognosis in a given subject.

## 3. Materials and methods

### 3.1. General subject data

All data collected in this study was provided by the Alzheimer's Disease Neuroimaging Initiative, a longitudinal multicenter research study, in collaboration with the Laboratory of Neuroimaging at the University of Southern California, designed to develop genetic, imaging, clinical, and biochemical biomarker data for AD (https://adni.loni.usc.edu/). Through a $60 million public-private partnership, ADNI researchers at 63 sites in the US and Canada carefully tracked the progression of AD in several subjects' brains using standardized protocols, allowing comparisons to be made between results based on ADNI's data. For this study, ADNI provided the Positron Emission Tomography (PET) scans; Mini-Mental State Exam (MMSE) scores; Functional Activities Questionnaire (FAQ) scores; Apolipoprotein (APOE) indication; age, gender, and weight classification that were used in this analysis. ADNI provides biomarker, imaging, clinical, and genetic data across three different groups: CN, MCI, and AD. PET scans, MMSE scores, APOE gene indication, FAQ scores, age, gender, and weight were collected for 1,298 individuals and 2,980 total scans across the amyloid cohort. There were subjects in this cohort that took at least one PET scan. Out of the 1,298 individuals from the amyloid cohort, 574 individuals were females and 646 were males. Subject information is shown in Table 1.

**Table 1:** ADNI subject information. All statistics were created from the set of unique patients (n = 1,298). Some attributes were missing from certain patients; 118 APOE A1 gene indications, 118 APOE A2 gene indications, 1,103 MMSE scores, and 224 FAQ scores were missing.

**Attributes**	**Value**					
Gender	Male			Female		
	646			574		
Mean age (years)	73.650					
Mean weight (kg)	75.276					
Amyloid positivity (%)	Positive			Negative		
	51.644%			48.366%		
Diagnosis	AD		MCI	SMC		CN
	172		633	101		392
APOE gene	APOE A1			APOE A2		
	ε2	ε3	ε4	ε2	ε3	ε4
	120	955	105	2	673	505
Mean FAQ score	3.985					
Mean MMSE score	26.605					

### 3.2. Imaging information and SUVR acquisition

The subjects in the amyloid cohort had the Florbetapir (18F-AV-45) injection for a PET protocol: 370 MBq (10.0 mCi) ± 10%, 20 min (4 × 5 min frames) acquisition at 50–70 min post-injection.

For each subject, all scans were collected from ADNI's image and data archive using a specific advanced search (“AV45 Coreg, Avg, Std Img and Vox Siz, Uniform Resolution”). The scans from this search were coregistered PET-MR and intensity normalized images that used Statistical Parametric Mapping (SPM8), a medical imaging process which allows SUV comparisons within select regions to be made in a given subject (Smith et al., 2022). Coregistering is important because MR has fine anatomical detail and PET cannot delineate anatomic structures (Robertson et al., 2016). PET transfers radiotracer information to MR throughout the coregistering process. Over the 20-min acquisition time, each image was resized to a uniform voxel size and each uniform size was 160 × 160 in-plane, along with 96 axial slices (Reith et al., 2020; Landau et al., 2021). All images were normalized and rescaled to 224 × 224 to accommodate the ImageNet pretraining.

We obtained the ^18^F-florbetapir cortical summary SUVR (“SUMMARYSUVR_WHOLECEREBNORM”) for each scan from the UC Berkeley AV45 Analysis. This calculation required FreeSurfer processing which included skull-stripping, segmentation, and delineation of cortical and subcortical regions in MRI scans which were co-registered to PET scans using SPM8. The cortical summary region (“COMPOSITE_SUVR”) was calculated by taking the mean uptake of all SUVR values from the subregions. These SUVR (“COMPOSITE_SUVR”) values were calculated with respect to the reference region (“WHOLECEREBELLUM_SUVR”) to derive the summary SUVR value for the whole cerebellum (“SUMMARYSUVR_WHOLECEREBNORM”) for each scan (Landau et al., 2021).


$$SUV\left(t\right)=\frac{c_{img}\left(t\right)}{ID/BW}$$

SUV(t) represents the radioactivity concentration in the subcortical and cortical regions (ROI) averaged during a period of time over the quantity of the injected dose (kBq/mL) divided by the weight (kg). This value is then calculated with respect to the reference region which determines SUVR.

### 3.3. Clinical data

An individual's age, gender, and weight were included in the clinical data for this analysis. Each individual in the ADNI dataset received a Mini-Mental State Exam (MMSE) after their testing session. CN or MCI subjects normally score between 24 and 30 inclusive while AD subjects normally score between 20 and 26 inclusive, showing that subjects who score lower than normal on this exam have cognitive impairment which is an indicator of Alzheimer's (Petersen et al., 2010).

Individuals also took a Functional Activities Questionnaire (FAQ) after their testing session. FAQ tests subjects with daily activities; the questionnaire has a range of 0–30 and subjects with a score of 6 or greater is suggestive of functional, cognitive impairment (Marshall et al., 2015).

Apolipoprotein E is a multifunctional protein with three isoforms: APOE ε2, APOE ε3, and APOE ε4. APOE ε4 has the possibility of forming stable complexes with Aβ peptides and it enhances Aβ aggregation (Huang and Mahley, 2014). This suggests that there is a correlation between APOE ε4 and pathogenesis of AD (Huang et al., 2017). While APOE ε4 is more of a genetic risk factor of AD, subjects with APOE ε3 are generally neutral and subjects with APOE ε2 are protective (Huang et al., 2017).

### 3.4. Deep learning implementation

The deep learning was implemented using TensorFlow (https://www.tensorflow.org/). The data was split into training (80%, *n* = 2,384), and testing (20%, *n* = 596) subsets to isolate training and testing results. The training set is a portion of the dataset that the model uses to fine tune weights while the testing set uses a separate portion of the dataset to evaluate real world performance of the model. Adam was used to optimize loss *via* backpropagation (Kingma et al., 2014), which works by dynamically adjusting the movement of the gradient to better optimize training. An initial learning rate of 0.001 was used with a batch size of 32 and a total of 20 epochs. All models were pre trained on ImageNet weights which were trained on the ImageNet dataset of 14 million natural images and 1,000 various classes. All images were modified to 224 × 224 pixels for ImageNet.

We first used the ResNet convolutional neural network (CNN) architecture, which solves the vanishing/exploding gradient problem *via* skip connections (He et al., 2015). Skip connections calculate the identity function of an earlier layer output and add it to the output value of the succeeding layer, preserving the gradient (Adaloglou, 2020). This occurs because the skip connection prevents the gradient from exploding or vanishing while retaining the progression through the layers. We tested this architecture using both ResNet-50, a 50-layer model of ResNet, and ResNetRS-50, a modern revision of the original ResNet architecture that achieves better computational efficiency by increasing image resolution more slowly and scaling model depth in overfitted areas (Tsang, 2022).

We also tested the EfficientNet CNN architecture which uses compound model scaling, a method which consistently relates resolution, depth, and width to each other (Tan and Le, 2021). EfficientNet uses a specific set of scaling coefficients to uniformly scale the resolution, width, and depth in order to achieve this constant ratio (Sarkar, 2021). The compound model scaling equation is ***α***•***β***^**2**^***•γ***^**2**^**≈ 2** where *α* represents depth, β represents width, and *γ* represents resolution. We tested the EfficientNet architecture using EfficientV2, a model which optimizes progressive learning of images to decrease overfitting and minimize the loss function, making it more efficient and accurate than EfficientNet while using less memory (Ibrahim, 2021).

Lastly, we tested the RegNet CNN architecture using the X002 variant. This architecture has significantly less parameters than the other CNN models, making the RegNet model more practicable for imaging tasks since it's less computationally intensive. RegNet uses self-regulation, a regulatory module which extracts spatio-temporal information from the intermediate layers of the network (Xu et al., 2021). In addition, RegNet is scalable, flexible, and efficient due to its weight residual connections, batch normalization, and regularization mechanism techniques (Xu et al., 2021).

### 3.5. Gradient boosted decision trees

Gradient Boosted Decision Trees sequentially build simple prediction models while constantly correcting the preceding model. This process improves the mistakes of the previous learner while simultaneously filtering out the correct observations (Gaurav, 2022). LightGBM is an open-source library that provides automatic feature selection and larger gradients which improves predictive performance of gradient boosted decision trees (Brownlee, 2021).

The GBM (Gradient Boosting Machine) was trained for 50,000 iterations with an early stopping sensitivity of 500 iterations. A random grid search was used to find the optimal hyperparameters for the GBM, by substituting random parameters and evaluating which parameters performed the best. Random state variables were never tested, with the intent to preserve scientific integrity.

### 3.6. Prediction approaches

Several prediction approaches were used with the data and the four architectures, ResNet-50, ResNetRS-50, EfficientNetV2-S, and RegNet. Binary classification and linear regression were performed on all four models' with either one or three slices of the brain from each subject. For single slice prediction, slice 48 was chosen out of the 96 axial slices, as it covers the central region of the brain which Alzheimer's often affects. Triple slice prediction used slices 36, 48, and 60, three areas of the brain with high amyloid burden. The proposed cutoff value of 1.11 for SUMMARYSUVR_WHOLECEREBNORM was used (Landau and Jagust, 2015).

First, all four networks were used to perform binary classification for single and triple slice on both the train and test set. Binary classification can be useful in determining positivity of Alzheimer's, although it lacks to precision of an exact SUVR value. In each model the average pooling layer precedes the fully connected layer with many activations. The fully connected layer was changed to down sample the activations in each model to 2 classes through linear down sampling. GlobalMaxPooling, preceding the final layer, was used to reduce spatial dimensions in the input data. The final layer was the sigmoid activation function which maps the input values from a range of 0 to 1. Binary Cross Entropy was used as the loss function:


$$L_{BCE}=-\frac{1}{n}\sum_{i=1}^{n}(Y_{i}\cdot \log{\hat{Y}}_{i}+(1-Y_{i})\cdot \log(1-{\hat{Y}}_{i}))$$

The ROC (Receiver Operator Characteristic) curve was used to show the performance of binarized classification. The ROC curve plots the true positive rate, the number of true positive results divided by the total number of positive cases, against the false positive rate, the number of false positive results divided by the total number of negative cases. A higher true positive rate to false positive rate ratio signifies a higher performing model. The area under the ROC curve (AUC) was used to measure the performance of each model.

**Figure 1:**
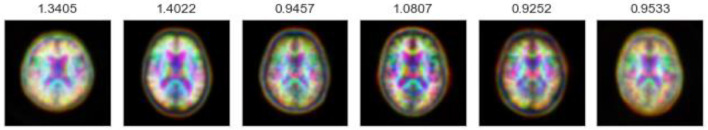
Color composites of various subjects with ground truth SUVR values.

Second, regression to SUVR was performed with three slices, slice 36, 48, and 60. Color composites were created by overlapping slices 36, 48, and 60 into the R, G, and B color channels respectively. Since the images are all black and white (thus governed by one color channel), no imaging information was lost by doing this, and during the prediction, the model will split the image into their respective color channels regardless, effectively providing three images worth of information in one. The linear activation function was used to downsample the activations of the global pooling layer into one output. Examples are shown in Figure 1.

**Figure 2:**
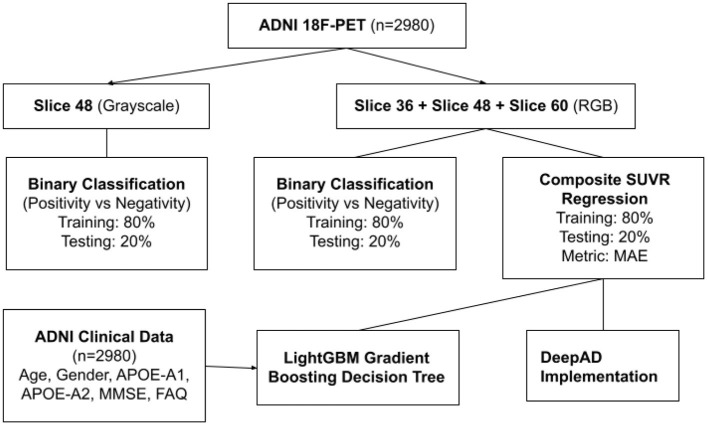
Flowchart of ADNI data applications.

For regression, the last fully connected was changed to one output which is linear only. Mean Absolute Error (MAE) was used to measure regression loss. MAE is the average difference between predicted and ground truth values, used in order to quantify the average difference between a patient's true SUVR value in the field vs. the model prediction.


$$\text{MAE}=\frac{\sum_{i=1}^{n}\left|y_{i}-x_{i}\right|}{n}$$

Finally, the best performing architecture was once again trained on the RGB color composites. The last fully connected layer was then removed, and the activations were extracted from the GlobalAveragePooling layer. The activations, as well as the clinical and genetic data, were fed into the Gradient Boosted Decision Tree, which then performed regression to reach a SUVR value. In effect, the linear layer was being replaced by GBDT functions, which has been shown to be more accurate (Ke et al., 2017). The basic model path is shown below.

## 4. Results

### 4.1. Binary classification

**Figure 3:**
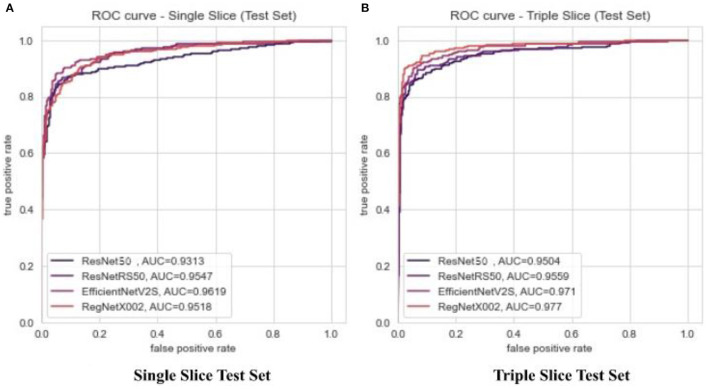
ROC and AUC for binary classification of ResNet-50, ResNetRS-50, EfficientNetV2-S, and RegNet-X002. **(A)** Single slice test set ROC. **(B)** Triple slice test set ROC.

First, we trained on binarized amyloid classification for SUVR (positive/negative), found using the cutoff value discussed above. Through preliminary tests, we settled on pre-trained ImageNet weights, 20 epochs, a batch size of 32, and an initial learning rate of 0.001 across all four regression models. The CrossEntropy losses for training and testing set after 20 epochs were 0.0 and 0.444, respectively. We used the ROC curve and AUC to evaluate the significance of binarized classification for each regression model. The results for all models for training and test sets for a variety of metrics are shown in Table 2. The ROC curve and AUC results are shown in Figure 3.

**Table 2:** Accuracy and precision of ROC curve results for ResNet-50, ResNetRS-50, EfficientNetV2-S, and RegNet-X002. Accuracy and precision were calculated from the ROC AUC curves of all four regression models. Accuracy refers to the percentage of correctly classified instances while precision refers to the percentage of positive predictions that were truly correct.

**Model architecture**	**Training**				**Testing**			
	**Single slice**		**Triple slice**		**Single slice**		**Triple slice**	
	**Accuracy**	**Precision**	**Accuracy**	**Precision**	**Accuracy**	**Precision**	**Accuracy**	**Precision**
ResNet-50	84.98	89.93	81.59	85.25	88.09	94.49	88.42	89.58
ResNetRS-50	89.22	90.74	92.24	95.11	90.60	94.46	92.79	97.39
EfficientNetV2-S	95.55	95.96	96.85	96.83	91.11	91.50	92.11	89.31
RegNet-X002	93.92	94.66	95.93	96.38	91.28	95.54	94.13	94.86

### 4.2. Amyloid regression model

**Figure 4:**
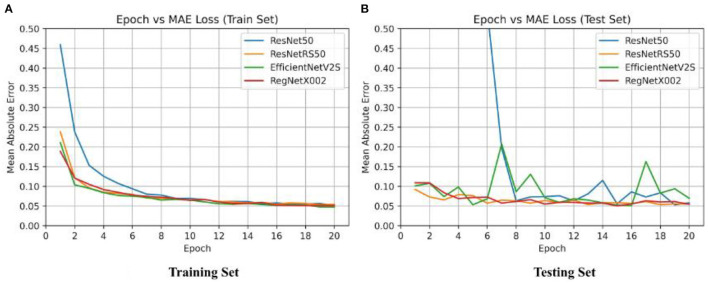
Epoch vs. loss for training and testing sets of ResNet-50, ResNetRS-50, EfficientNetV2-S, and RegNet-X002. **(A)** Training set epoch vs. MAE. **(B)** Testing set epoch vs. MAE.

**Figure 5:**
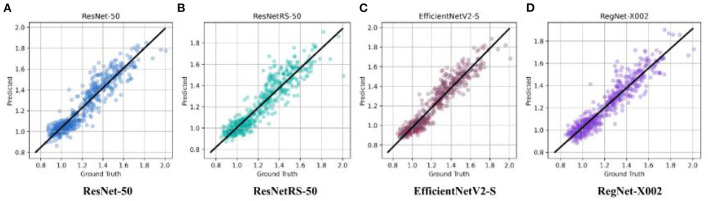
Ground truth vs. predicted SUVR values for ResNet-50, ResNetRS-50, EfficientV2-S, and RegNet-X002. **(A)** ResNet-50. **(B)** ResNetRS-50. **(C)** EfficientNetV2-S. **(D)** RegNet-X002.

After binary classification, regression was used to predict an exact SUVR quantity. For regression we used 20 epochs, and an initial learning rate of 0.001. The same network architectures used for binary classification were used for linear regression. The MAE loss for the training set and testing set of the four regression models are shown in Figure 4. Ground truth versus predicted SUVR for each regression model is shown in Figure 5.

The best results were achieved on the RegNet architecture. RegNet-X002 was the smallest of all the models with only 2,337,009 parameters, yet outperformed substantially compared to more costly models, such as ResNetRS-50. Additionally, the difference between the train set and test set accuracy was lowest in the RegNet model, signifying less overfitting. The MAE loss results for all four regression models are shown in Table 3.

**Table 3:** MAE loss results for ResNet-50, ResNetRS-50, EfficientNetV2-S, and RegNet-X002. Each trial was run a single time and results were taken (n = 2,980).

**CNNs**	**MAE for train set**	**MAE for test set**	**Parameters**
ResNet-50	0.0380	0.0665	23,589,761
ResNetRS-50	0.0543	0.0550	33,698,337
EfficientNetV2-S	0.0475	0.0696	12,932,159
RegNet-X002	0.0513	0.0542	2,337,009

Minimal consistency improvements were made after 20 epochs, thus we decided to train for 20 epochs in succeeding tests. The RegNet architecture performed the best out of all the models. Table 3 shows that RegNet-X002 achieved an MAE loss of 0.0542 which was much lower than the other three regression models. Because of this, RegNet was the architecture that was used with the Gradient Boosted Decision Tree.

### 4.3. Amyloid regression and gradient boosted decision tree model

Since RegNet was the highest performing model, we used this architecture for regression and gradient boosted decision trees. We used the RegNet-X064 variant instead of X002 since the increase in capacity, layers, and overall size of the network could impact predictive performance. With 24,660,089 parameters, the RegNet-X064 regression model used 20 epochs and an initial learning rate of 0.001. GlobalAveragePooling was the layer preceding the fully connected layer, outputting 1,624 activations per subject in the RegNet-X064 architecture. This regression model achieved an MAE loss value of 0.0278 for the training set and 0.0461 for the testing set, already a significant improvement from all previous models.

We then used the LightGBM library for our GBDT because it has improved predictive performance compared to other GBDTs. Using LightGBM, we inputted clinical data such as FAQ MMSE scores, and APOE gene indication. We also used random grid search for 100 iterations to find the best hyperparameters for the GBDT model which resulted in the lowest MAE loss. The optimal hyperparameters were a max depth of 9, a feature fraction of 0.5, and a learning rate of 0.045. With RegNet-X064 and LightGBM, this model achieved an MAE loss of 0.000000444 for the training set and 0.0441 for the testing set, outperforming all previous configurations by a high margin.

**Figure 6:**
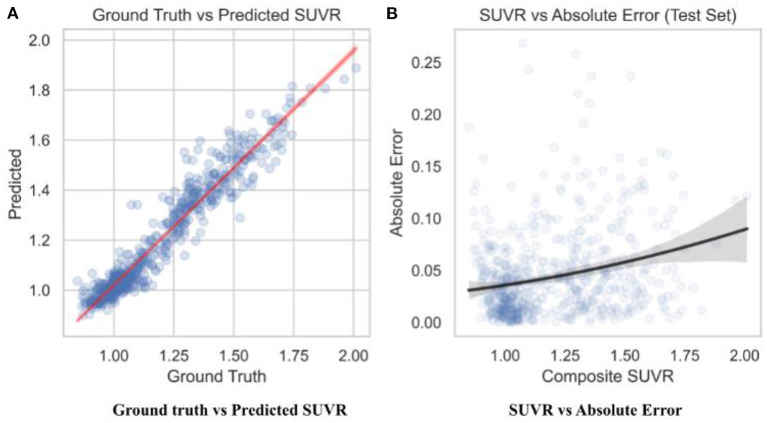
Predicted SUVR vs. various variables. All data shown was taken from the test set only (*n* = 596). **(A)** RegNet + GBDT ground truth vs. predicted SUVR. **(B)** RegNet + GBDT SUVR vs. absolute error.

To observe the consistency improvements, a predicted vs. ground truth plot was drawn using the test set in Figure 6. The plot indicates the skew of MAE loss at different SUVR values, showing when the model predicts SUVR the best. The model shows little to no skew, with tighter error at the cutoff value of 1.11 and highest at 1.4.

The StatsModels library (https://www.statsmodels.org/) was used to calculate statistical significance between the predicted SUVR values in the test set and the ground truth SUVR values. The adjusted *R*^2^ coefficient of determination was calculated to be 0.997. *R*^2^ is the amount of variance explained by the linear model; 99.7% of data variance in the test set was explained. It is calculated through residuals: $R^{2}=1-\frac{RSS}{TSS}$, where RSS is the sum of squares of residuals and TSS is total sum of squares. The *p*-value was calculated to be statistically zero, which represents the probability that the relationship observed was due to chance variance. The probability f-statistic was also calculated to be statistically zero, which similarly represents the probability that a model with no independent variables would perform better than the observed model. The approaches used with the data are summarized in Figure 2.

### 4.4. Web application implementation

We implemented our prediction model on to a web application so that our diagnostic tool could be accessible by physicians and diagnosticians. The web application was developed on the Flask software platform. We used Heroku, a cloud-based web application service, to deploy our web application for public use. We named our web application DeepAD, since it provides an accurate SUVR calculation for Alzheimer's using our deep learning model. The web application is available at (https://deepad.herokuapp.com/).

For the web application we used the RegNet-X002 architecture. Although RegNet-X064, which had a lower MAE loss than RegNet-X002, would have been the preferred model for the web application, our web application service, Heroku, had a hard bandwidth issue due to limited ram which prevented us from using RegNet-X064. We were also unable to use LightGBM for our web application model, preventing us from using clinical data. However, this was only a minor issue since clinical data holds a small factor in SUVR prediction. Also, since diagnosticians and doctors would already be assessing a patient's cognitive state with clinical data, it would be illogical to also add clinical data to the web application model.

## 5. Discussions

Several explanations can be provided for the best achieved MAE loss. Before testing regression, binary classification amyloid positivity and negativity. Across all four architectures, the AUC of the ROC curves show that each network had a higher accuracy with triple slice compared to single slice. From single to triple slice, ResNet-50's accuracy improved by 1.91%, ResNetRS-50's accuracy improved by 0.12%, EfficientNetV2S's accuracy improved by 0.91%, and RegNet-X002's accuracy improved by 2.52%. Therefore, three slices were used for linear regression.

For linear regression, RegNet-X002 achieved the lowest MAE loss of 0.0542 compared to the other architectures. RegNet-X002 had a small difference of 0.0029 between the train and test set, signifying less overfitting in the model. In addition, the regression plots show that RegNet-X002 performed the best at the SUVR value 1.1, the cutoff between an amyloid negative and amyloid positive subject. This signifies that the model performs the best when it's most important, which is when a patient could be borderline for AD diagnosis. This seems to suggest that RegNet's self-regulation and normalization techniques allowed the model to achieve higher accuracy than the other CNN architectures, along with being the most computationally efficient model.

After determining that RegNet was the best network architecture, we used the X064 variant for the RegNet architecture. To achieve better model performance, we implemented LightGBM, a gradient boosted decision tree. The addition of clinical data, such as MMSE scores, FAQ scores, and APOE indication, slightly helped the model accuracy. Overall, the RegNet-X064 and LightGBM model achieved an MAE loss of 0.0441 for the testing set which outperformed any other CNN tested in this study.

The test results were statistically significant on the best performing configuration. Based on the *p*-values, the null hypothesis that there is no inherent relationship between the model's predictive capability and the ground truth values can be safely rejected, as the *p*-value of 0.0 is less than the 0.05 threshold. The *R*^2^ coefficient of determination indicated that only 0.3% of the predictions on the test set had variance that couldn't be explained, signifying that the model's prediction is reliable.

Results from our study show that the use of axial slices 36, 48, and 60 per subject, MMSE scores, FAQ scores, APOE indication, and LightGBM paired with RegNet-X064 improved the linear regression model's prediction performance of SUVR significantly. Our best regression model (0.0441 MAE loss) achieved an accuracy of 96.4% over the range of SUVR values. Compared to the study conducted by Kim et al. (2019), our MAE loss was lower by 0.0159, signifying a large increase in the predictive performance of our model compared to their model. We achieved a better MAE loss since our model was more powerful, had better hypter-parameter tuning, and included clinical data with LightGBM.

Although the calculation of SUVR for a given subject provides the uptake quantification of the radiotracer (18F-AV-45) based on the accumulation of amyloid, this calculation approach is inefficient and less accurate compared to a deep learning approach for calculating SUVR based on coregisterd PET-MR images. When comparing SUVR prediction performance from a linear regression model to SUVR calculations by readers, Reith et al. (2020) found that the three SUVR readers took 24:28 min for 100 test samples. Our implemented model for the web app, RegNet-X002, took ~3 s to process 596 samples while the SUVR readers would have taken ~145 min to calculate the SUVR values from our test samples with the same SUVR readers. Individual SUVR calculations are not ideal when diagnosing a patient with a ^18^F-florbetapir PET scan. Our proposed model solves the efficiency problem that SUVR readers experience when calculating SUVR values. To make our research easily accessible, we created a web application (DeepAD) to implement our proposed model. Although the RegNet-X064 model achieved the best performance, we developed the webapp using the RegNet-X002 architecture since its limited parameter count satisfied the constraints of Heroku, the platform for the webapp.

Noise in the ground truth SUVR calculations for each subject's scan needs to be considered with the result of the regression model. Reith et al. (2020) showed that each reader calculated the SUVR value at a different pace and accuracy which contributes to the SUVR variability factor. There was also noticeable noise in the ^18^F-florbetapir PET scans. The pixel count of 160 × 160 doesn't provide as much information compared to a pixel count with greater dimensions. There was noise in the chosen slices because there might have not been enough coverage for parts of the brain which have more present amyloid or are highly correlated to AD.

There are several limitations to consider in this study. Firstly, we were only able to examine the information in the input and the output layers of the CNN but not the middle layers which are responsible for tasks such as data transformation and automatic feature creation. For future use of this model, images fed as input data would require a specific process. Each ^18^F-florbetapir scan needs to be co-registered using Statistical Parametric Mapping (SPM8) to the same subject's MRI image. This process requires the subject to get a PET and MRI scan. Also, SPM8 software is necessary for the co-registering process. This process alone questions the fiscal practicality the imaging (Landau et al., 2021).

## 6. Conclusions

Ultimately, we used deep learning architecture and Gradient Boosting Decision Trees along with imaging, clinical, and SUVR data to construct a regression model that quantifies amyloid SUVR from ^18^F-florbetapir radiotracer uptake in PET scans. At least temporarily, our proposed model's efficiency could provide a supplement to the process of manually calculating SUVR. In addition, our model is computationally efficient, processing hundreds of samples within seconds. While there's still much research to be done, this model shows some promise for automated SUVR calculations, achieving similar accuracy compared to individual SUVR readers. Our proposed deep learning model is available on DeepAD, our web application which allows anyone to upload a PET-MR scan and receive a SUVR calculation. Future research should investigate more clinical indicators of AD, such as FAQ and MMSE scores, and analyze other protein deposition linked to Alzheimer's. Along with investigating new proteins indicative of Alzheimer's, new radiotracer biomarkers could be discovered to trace these new proteins. Future work can be done to improve the PET scan. Although PET scans are stochastic, improving PET spatial resolution and reconstructing algorithms which obtain imaging of a subject based on radiotracer distribution can limit variability in SUVR and noise in PET scans. Better PET imaging would also result in better accuracy for regression models when training the network.

## Data availability statement

The original contributions presented in the study are included in the article/supplementary material, further inquiries can be directed to the corresponding authors.

## Ethics statement

Ethical review and approval was not required for the study on human participants in accordance with the local legislation and institutional requirements. Written informed consent for participation was not required for this study in accordance with the national legislation and the institutional requirements. Written informed consent was obtained from the individual(s) for the publication of any potentially identifiable images or data included in this article.

## Author contributions

SM: conceptualization, methodology, data collection and processing, machine learning, hyperparameter tuning, data and model performance visualization, and paper editing. KD: conceptualization, data collection, methodology, disease background research, validation, and paper writing and editing. Both authors have read and agreed to the final version of the manuscript for publication.
